# Astrocytes Maintain Glutamate Homeostasis in the CNS by Controlling the Balance between Glutamate Uptake and Release

**DOI:** 10.3390/cells8020184

**Published:** 2019-02-20

**Authors:** Shaimaa Mahmoud, Marjan Gharagozloo, Camille Simard, Denis Gris

**Affiliations:** Program of Immunology, Department of Pharmacology-Physiology, Faculty of Medicine and Health Sciences, University of Sherbrooke, Sherbrooke, QC J1H 5N4, Canada; shaimaa.mahmoud@usherbrooke.ca (S.M.); Marjan.Gharagozloo@usherbrooke.ca (M.G.); Camille.Simard@usherbrooke.ca (C.S.)

**Keywords:** astrocytes, glutamate uptake, glutamate release, excitotoxicity, CNS

## Abstract

Glutamate is one of the most prevalent neurotransmitters released by excitatory neurons in the central nervous system (CNS); however, residual glutamate in the extracellular space is, potentially, neurotoxic. It is now well-established that one of the fundamental functions of astrocytes is to uptake most of the synaptically-released glutamate, which optimizes neuronal functions and prevents glutamate excitotoxicity. In the CNS, glutamate clearance is mediated by glutamate uptake transporters expressed, principally, by astrocytes. Interestingly, recent studies demonstrate that extracellular glutamate stimulates Ca^2+^ release from the astrocytes’ intracellular stores, which triggers glutamate release from astrocytes to the adjacent neurons, mostly by an exocytotic mechanism. This released glutamate is believed to coordinate neuronal firing and mediate their excitatory or inhibitory activity. Therefore, astrocytes contribute to glutamate homeostasis in the CNS, by maintaining the balance between their opposing functions of glutamate uptake and release. This dual function of astrocytes represents a potential therapeutic target for CNS diseases associated with glutamate excitotoxicity. In this regard, we summarize the molecular mechanisms of glutamate uptake and release, their regulation, and the significance of both processes in the CNS. Also, we review the main features of glutamate metabolism and glutamate excitotoxicity and its implication in CNS diseases.

## 1. Introduction

Astrocytes represent the majority of the cells in the central nervous system (CNS), and they cover the CNS in a continuous and non-overlapping manner [1]. This allows astrocytes to be omnipresent and perform crucial functions supporting neuronal activity and maintaining the homeostasis of the CNS [1]. Since early in ontogeny, they support migration of the progenitor cells and ensure normal function of the developing synapses. Through dual connections to neurons and blood vessels, astrocytes provide neurons with nutrients, remove the products of metabolism, control the amount of blood flow perfusing the CNS, and maintain the integrity of the blood-brain-barrier (BBB). Using their ionic and aquaporin-4 water channels, astrocytes control the ionic and fluid balance in the CNS [1], in addition to their innate immune response to any CNS injury or disease [2]. In summary, directly or indirectly, astrocytes fulfill all the functions of the CNS aside from electrical conduction.

One of the critical functions of astrocytes in the CNS is the regulation of neurotransmitter homeostasis, as they uptake synaptically-released neurotransmitters, such as glutamate, γ-aminobutyric acid (GABA) and glycine, metabolize them and release their precursors back to neurons [1]. This review will focus on the role of astrocytes in glutamate homeostasis. Although glutamate is the most common excitatory neurotransmitter in the CNS [3,4], the excess of glutamate in the synaptic and extra-synaptic space leads to neuronal hyperexcitation and subsequent neuronal death, in a process known as “glutamate excitotoxicity”, which accompanies several inflammatory and neurodegenerative diseases of the CNS [5]. Therefore, unused glutamate during synaptic transmission must be rapidly cleared from the extracellular space. The mission of glutamate clearance is achieved, primarily, by astrocytes and is mediated by glutamate uptake transporters [6].

On the other hand, recent studies revealed that, in addition to uptake, astrocytes release traces of glutamate to the adjacent neurons, which help to synchronize their firing and modulate their excitatory or inhibitory transmission [7]. This astrocytic glutamate release is, plausibly, mediated by Ca^2+^-dependent exocytosis, using similar machinery to that utilized by neurons in the synaptic glutamate release. Other mechanisms have also been proposed to mediate glutamate release from astrocytes [8]; however, it is yet a matter of debate whether astrocytes could release glutamate, in vivo, in healthy conditions.

Accordingly, by controlling the balance between glutamate uptake and release, astrocytes have the ability to maintain glutamate homeostasis, support normal neuronal function, and protect against glutamate excitotoxicity. In this review, we will summarize most of the current research concerning astrocytes’ roles in glutamate homeostasis, including their ability to uptake and release glutamate.

## 2. Glutamate Uptake in the CNS

Although the majority of CNS cells partake in the extracellular glutamate removal, astrocytes are, by far, the most efficient in this process, as they remove about 90% of all released glutamate in the CNS [9,10]. The main route of glutamate uptake is achieved through two types of glutamate transporters, Na^+^-independent and Na^+^-dependent transporters [6,11].

### 2.1. Glutamate Uptake Transporters

#### 2.1.1. Na^+^-Independent Glutamate Uptake Transporters

Na^+^-independent transporters are chloride-dependent antiporters that allow cystine/glutamate exchange [12]. Many cell types in the CNS express the Na^+^-independent transporters, including astrocytes [12,13,14], microglia [15], Müller cells in the retina [16], and glioma cells [17]. The main function of these transporters is to uptake cystine, which is a molecule utilized by most of the cells in the body to synthesize the intracellular antioxidant enzyme, glutathione. Although Na^+^-independent transporters possess the same or a slightly higher affinity to glutamate compared to N^+^-dependent transporters, they uptake a significantly lesser amount of glutamate under physiological conditions (less than 5% of the total extracellular glutamate) [6]. Theoretically, this percentage could be increased, only, if cystine uptake is inhibited, which is unlikely to occur in normal conditions as the cells will be under the risk of lethal oxidative stress [13,14].

#### 2.1.2. Na^+^-Dependent Glutamate Uptake Transporters

Na^+^-dependent transporters are known as excitatory amino acid transporters (EAATs), and they are responsible for the uptake of the majority of the extracellular glutamate [6,11]. Till now, five isoforms of EAATs were identified, EAAT-1, EAAT-2, EAAT-3, EAAT-4, and EAAT-5. The first 2 isoforms, EAAT-1 and EAAT-2, in human [18,19] are known in murine animals as glutamate-aspartate transporter (GLAST) [20] and glutamate transporter-1 (GLT-1) [21], respectively. Noteworthy, EAAT-1/GLAST and EAAT-2/GLT-1 represent the majority of EAATs and are expressed, mainly, by astrocytes. EAAT-3 (excitatory amino acid carrier-1; EAAC-1) is expressed by neuronal cell bodies [22]. EAAT-4 is expressed by cerebellar Purkinje cells [23] and the last one, EAAT-5, is, exclusively expressed in the retina [24]. In this review, we focus on EAAT-1 and EAAT-2 in astrocytes, as they are responsible for the uptake of 80–90% of the total extracellular glutamate in the CNS [9,10].

### 2.2. Expression Profile of EAAT-1 and EAAT-2

In 1997, Gegelashvili and Schousboe [25] reported that EAAT-1 and EAAT-2 (GLAST and GLT-1, respectively) have a similar structure, as they are 65% homologous at the amino acid level. Functionally, both have the same affinity to glutamate, with a K_m_ value ranging from 10 to 77 µM for EAAT-1 (GLAST) [25,26,27], and from 36 to 97 µM for EAAT-2 (GLT-1) [27,28]. Both are capable of reducing the extracellular glutamate to the same level [29]; however, they differ in their expression profile in the CNS.

Early after birth, EAAT-1/GLAST is expressed by radial glial cells and immature astrocytes in the forebrain and the cerebellum [30]. In adulthood, EAAT-1/GLAST is, predominantly, expressed in Bergmann glial cells in the cerebellum [31,32,33], Müller glia in the retina [34,35], the circumventricular organs [36], and in cochlear glial cells in the inner ear [37,38,39], while it acquires a limited expression in the forebrain. In adult CNS, EAAT-1/GLAST is expressed, mainly, by mature astrocytes [9,40] and, to a lesser extent, by microglia [41] and oligodendrocytes [41,42].

Contrariwise, EAAT-2/GLT-1 is not detected in rat brains early after birth, and its expression maximizes within 3 to 5 weeks of age [43,44,45]. After CNS maturation, EAAT-2 represents the major EAAT expressed by mature astrocytes in the CNS, except in the areas where EAAT-1 predominates [31].

Several reports showed a limited expression of both EAAT-1/GLAST and EAAT-2/GLT-1 in primary neuronal cultures [46,47,48]. GLT-1 was detected in the developing axons of mice spinal cords [49] and neurons after hypoxic-ischemic injury [50].

In astrocytes, EAAT-1 and EAAT-2 are mostly distributed in clusters on the peri-synaptic astrocytic processes in contact with active synapses of glutamatergic neurons [51,52,53,54,55,56]. While the expression of EAAT-1 in the adult CNS is relatively constant, the expression of EAAT-2 positively correlates with synaptic activity [57] and is upregulated with increased glutamate release from the surrounding glutamatergic neurons [54,58].

### 2.3. EAAT-2 and EAAT-1 in Astrocytes Play the Major Role in Glutamate Uptake in the CNS

In 1989, Rosenberg and Aizenman reported that in astrocyte-poor cultures of the rat cerebral cortex, there was a 100-fold increase in neuronal death caused by glutamate neurotoxicity [59]. Later, in vivo studies in hippocampal slices [60,61,62] and cerebellar preparations [63,64] showed that synaptic glutamate release from excitatory neurons induced glutamate transporter-associated currents in astrocytes, while no similar currents were detected in neurons [65]. Surprisingly, these transporter-associated currents in astrocytes were reduced in slices prepared from GLT-1 knockout mice or after the use of dihydrokainate (DHK, a GLT-1 selective inhibitor) and this effect was similar to the results obtained with the use of d,l-threo-β-hydroxyaspartate (THA, a nonselective EAAT inhibitor) [62].

In animal models, GLT-1 and GLAST deficient rats developed neurodegeneration and progressive paralysis [66]. Moreover, studies using GLT-1 knockout mice further demonstrated the preferential importance of this transporter in glutamate uptake, as these mice experienced an increased incidence of lethal seizures and demonstrated a high susceptibility to neuronal loss [67]. Interestingly, the same phenotype of GLT-1 knockout mice has been replicated in mice lacking GLT-1, exclusively, in astrocytes in a conditional knockout mouse model [68], confirming the specific importance of astrocytes in the process of glutamate uptake. Besides GLT-1, in 1998, Watase et al. [69] reported that GLAST mutant mice developed motor incoordination and cerebellar injury. In contrast, EAAC-1 (EAAT-3) [66,70] and EAAT-4 [71] deficient mice experienced only minor neurological deficits, while no EAAT-5 knockout animal model has been reported.

**Figure 1:**
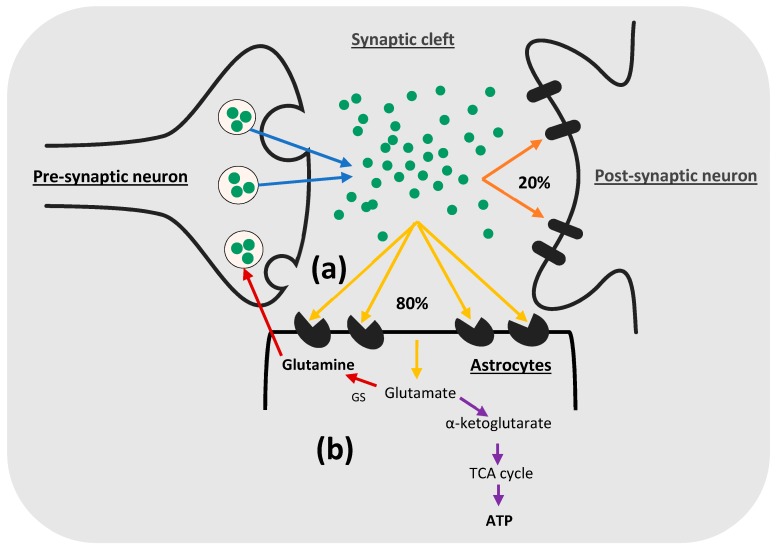
Glutamate uptake and metabolism by astrocytes: (**a**) Glutamate uptake by astrocytes: After release of glutamate from the presynaptic neurons (blue arrows), only 20% of glutamate is taken up by post-synaptic glutamate receptors to transmit excitatory impulses (orange arrows), while astrocytes uptake most of the remaining glutamate by their glutamate uptake transporters, EAAT-1 and EAAT-2 (yellow arrows), which are expressed on the surface of the astrocytic peri-synaptic processes; (**b**) glutamate metabolism in astrocytes: In astrocytes, glutamate could be metabolized to glutamine by glutamine synthetase (GS) (red arrows), then glutamine is released to the extracellular space to be taken up by neurons and used to resynthesize glutamate or GABA. On the other hand, glutamate could be oxidatively metabolized to α-ketoglutarate, which is used in ATP synthesis (violet arrows).

In the CNS, as soon as glutamate is released from the presynaptic neurons, a small percentage of this glutamate (20% in the retina and cerebellar cortex and, probably, a lesser percentage in the hippocampus) is taken up by postsynaptic neuronal receptors [65,72,73], while the majority of this synaptically-released glutamate (80% or more) diffuses out of the synaptic cleft and is cleared from the extracellular space by astrocytic EAAT-1 and EAAT-2 (Figure 1a) [62,65]. Anderson and Swanson presented evidence that astrocytes are the key players in the glutamate uptake process [6], as astrocytes have a greater ability than neurons to conserve the driving forces for glutamate uptake. They maintain a more stable membrane potential with high extracellular Na^+^ and low K^+^ compared to neurons, which have a less stable Na^+^/K^+^ ratio, caused by repeated neuronal firing. Astrocytes also provide a sufficient supply of adenosine triphosphate (ATP), necessary for glutamate uptake, even in the case of hypoxia or glucose deprivation [74,75,76,77]. Moreover, the rapid conversion of glutamate to glutamine in astrocytes ensures a low level of intracellular glutamate, which creates a low threshold across the astrocyte plasma membrane that stimulates glutamate uptake, while neurons maintain a high level of intracellular glutamate, which decreases their ability to uptake glutamate [78,79,80,81,82,83]. In addition, glutamine formed in astrocytes is utilized by neurons as a precursor to resynthesize active neurotransmitters, such as glutamate and GABA, which makes glutamate uptake by astrocytes necessary to complete this glutamate/GABA-glutamine cycle [82,84,85].

### 2.4. Mechanism of Glutamate Uptake by EAATs

Normally, glutamate concentration in the extracellular space is in the micromolar (µM) range [86], while its intracellular concentration is in millimolar (mM) quantities [87]. Therefore, astrocytes uptake glutamate against its concentration gradient. Many electrophysiological studies describe the stoichiometry of glutamate uptake derived by the inward transport of 3 Na^+^ and 1 H^+^ ions with each glutamate anion in exchange with the outward transport of 2 K^+^ ions, in respect to their concentration gradients [88,89]. Hence, the process of glutamate uptake is accompanied by a change in the astrocyte membrane potential caused by the inward transport of two extra positive charges, creating a form of membrane depolarization known as an “uptake current”, which was first described by Bowman and Kimelberg in 1984 [90]. Moreover, glutamate uptake is associated with the transport of Cl^−^ and H^+^ [91,92] in addition to many other ions, which causes changes in the ionic concentrations in astrocytes. Although these ionic changes do not seem to influence glutamate uptake, they are essential for the maintenance of the ionic balance in astrocytes [93].

Glutamate uptake is considered one of the highest energy-consuming processes in the CNS. To uptake glutamate against its concentration gradient, astrocytes require high levels of energy, which is more than 1 ATP molecule for each molecule of glutamate taken up [94]. Noteworthy, the activation of Na^+^/K^+^ ATPase in astrocytes to breakdown ATP [95,96] enhances the processes of glycolysis and glycogenolysis [97,98,99] and results in lactate formation, which is released from astrocytes into the extracellular space to be used by neurons as a source of energy [100,101].

### 2.5. Metabolism of Glutamate in Astrocytes

Glutamate can be metabolized in astrocytes by one of two major pathways (Figure 1b). The majority of glutamate is converted to glutamine [102] by glutamine synthetase enzyme, preferentially expressed in astrocytes and, to a lesser extent, in oligodendrocytes, but not in neurons [103,104]. Then, glutamine is released from astrocytes to the extracellular space by electroneutral, sodium-dependent transporter, SN1 [105,106,107], following which glutamine is taken up by the system A transporters into neurons [106]. In neurons, glutamine serves as a precursor for the synthesis of active neurotransmitters, such as glutamate or GABA, which are packed inside vesicles to be released again during synaptic transmission [82,84,85]. Furthermore, activation of glutamine synthetase has a detoxifying effect, as it breaks down the blood-derived ammonia and brain ammonium (NH_3_/NH_4_^+^) [108,109], keeping their concentration in the brain below 0.1 mM [110]. Therefore, astrocyte dysfunction often leads to the accumulation of (NH_3_/NH_4_^+^) in the brain, causing ammonium neurotoxicity [111,112].

A significant proportion of glutamate is oxidatively metabolized to the tricarboxylic acid (TCA) intermediate, α-ketoglutarate, which serves as a substrate for ATP production [113]. Complete oxidative degradation of glutamate is achieved, primarily, by oxidative deamination, catalyzed by the mitochondrial enzyme, glutamate dehydrogenase (GDH), and this process expands the pool of the TCA cycle intermediates [78]. The partial oxidative metabolism of glutamate is achieved by the transamination via aspartate aminotransferase (AAT), alanine aminotransferase, and branched chain aminotransferase (BCAT) enzymes [79]. Noteworthy, oxidative metabolism of glutamate results in the production of an enormous amount of ATP in astrocytes, which exceeds the ATP required for glutamate uptake [114].

The preference between these two metabolic pathways depends, mainly, on the concentration of glutamate in the extracellular space [6]. If it is less than 0.2 mM, glutamate is metabolized to glutamine to provide glutamate to neurons, while the oxidative metabolism is favored if the extracellular glutamate concentration is higher than 0.2 mM to provide the required energy for glutamate uptake [80,81].

### 2.6. EAAT-1 and EAAT-2 Regulation of Expression

Many factors regulate the transcription, translation, and post-translational modification as well as the transporter activity of EAAT-1 and EAAT-2 under physiological and pathological conditions (extensively reviewed elsewhere) [25,115,116]. Here, we summarize the key regulatory factors at different levels.

#### 2.6.1. Transcriptional and Translational Modifications

One of the major factors that influence the expression of glutamate transporters is the concentration of glutamate in the extracellular space. In this context, in 1996, Gegelashvili and colleagues reported that incubating astrocytes with l-glutamate in astrocyte cell cultures increased the expression of GLAST protein, while it did not have any effect on its mRNA level. This effect of glutamate on GLAST was blocked by inhibitors of AMPA/Kainate receptors [117]. In line with these results, Duan et al. reported that l-glutamate also upregulates the trafficking of GLAST protein to the cell surface and this effect was not blocked by inhibitors of protein kinase A (PKA), protein kinase C (PKC), or phosphoinositide 3-kinase (P13K) [118].

Despite many attempts, very little is known about the molecular mechanisms and signaling pathways involved in the trafficking of GLAST and GLT-1 to the cell membrane. Therefore, identification of these molecules, in the future, will be of great significance, as it will allow us to design new and fast-acting therapies that directly target cellular localization of GLAST and GLT-1, with no requirement of protein synthesis.

In addition to glutamate, stimulation of glutamate receptors also plays a role in the expression of astrocyte glutamate transporters. Activation of group II metabotropic glutamate receptors (mGluRs) was shown to enhance GLAST mRNA and protein expression [119,120], by activation of the ERK/P13K/ NF-κB pathway [121], while activation of group I mGluRs inhibits both GLAST and GLT-1 expression [120] via the same signaling pathway [121]. Likewise, the use of ionotropic glutamate receptor (iGluR) agonists activates the PKC signaling pathway and results in downregulation of GLAST expression [122,123].

It is well known that astrocytes in cell cultures express GLAST exclusively with no or very minimal GLT-1 expression; however, the expression of GLT-1 can be induced by co-culturing astrocytes with neurons or neuron-conditioned medium (NCM) [25,57,124,125]. This NCM-induced GLT-1 expression was completely blocked by inhibiting PI3K, tyrosine kinase, and NF-κB pathways [57,126]. One of the soluble molecules that could be mediating this effect of NCM on GLT-1 is a neuron-derived peptide known as the pituitary adenylate cyclase-activating polypeptide (PACAP), and its effect on GLT-1 was inhibited by blocking both the PKA and PKC pathways [127]. Regarding its effect on GLAST, PACAP increases its protein expression and its maximum velocity of glutamate uptake via activation of the PKA signaling pathway [128].

In another study, treating astroglial cultures with dibutyryl cyclic adenosine monophosphate (dbcAMP) induced upregulation of both GLT-1 and GLAST mRNA and protein expression as well as their uptake activity; however, this effect on both transporters was not mediated by protein kinase A (PKA) [58].

Many growth factors upregulate GLAST protein and mRNA levels, including epidermal growth factor (EGF), glial cell line-derived neurotrophic factor (GDNF), basic fibroblast growth factor (bFGF), and insulin-like growth factor-1 (IGF-1) [127,129,130]. On the other hand, EGF and TGF-α increase GLT-1 mRNA and protein expression as well as its uptake activity through activation of P13K and NF-κB pathways [126].

Hormones, such as estrogen, and a synthetic estrogen receptor modulator, Tamoxifen, induce upregulation of both GLAST [131,132] and GLT-1 [131,133,134] mRNA and protein levels in primary astrocyte cultures. Another group of hormones, glucocorticoids, were reported to upregulate GLT-1 mRNA and protein expression [135], while, in contrast, insulin has been reported to downregulate GLAST expression [136].

Curiously, GLT-1 expression can be induced in astrocytes by treating astrocyte cell cultures with ATP or adenosine. This effect is mediated by P2Y (ATP receptors) and A1 (adenosine receptors), respectively, through activation of the ERK/NF-κB signaling pathway [137,138]. Extracellular ATP also induces the formation of GLAST clusters on the astrocyte cell membrane, and this effect is, apparently, mediated through P2 receptors [139].

Both GLAST and GLT-1 protein expression is inhibited by endothelins [140,141] via activation of the PKA signaling pathway. Similar effects of dopamine [142] and retinoic acid [143] were revealed exclusively on GLT-1.

In pathological conditions, Tumor necrosis factor-α (TNF-α), which is a pro-inflammatory cytokine, has been reported to decrease the expression of both GLAST [144] and GLT-1 proteins, through activation of the NF-κB and YY1 transcription factors [145,146].

In 1995, Torp et al. reported that transient ischemia downregulates GLT-1 mRNA and protein levels [147]. Two recent studies demonstrated that GLAST activity is also downregulated in hypoxic conditions, through repression of the JAK/STAT signaling pathway [148,149].

From the previous reports, it is clear that many signaling pathways, such as the NF-κB, PKA and PKC, are involved in the regulation of EAAT-1/GLAST and EAAT-2/GLT-1 expression. Depending on the interaction with various environmental factors and signaling pathways, the activation of these pathways could mediate stimulation or repression of glutamate transporter expression.

#### 2.6.2. Post-Translational Modifications and Regulation of the Transporter Activity

Following protein synthesis and during its maturation, GLAST and GLT-1 proteins express potential sites of glycosylation in the extracellular domain. Interestingly, two studies, using an overexpressing non-glycosylated form of GLAST in *Xenopus oocytes* [150] and a non-glycosylated form of GLT-1 in BHK cells [151], reported that protein glycosylation had no effect on either the movement of the transporters to the plasma membrane or on their ability to uptake glutamate.

In 2004, Butchbach et al. demonstrated that the interaction of EAAT-2 with membrane cholesterol is necessary for stabilization of the transporter protein on the cell plasma membrane. When cholesterol was removed, rapid internalization of EAAT-2 by endocytosis occurred and resulted in reduced glutamate uptake in primary astroglial cultures [152].

Many contradictory results were published concerning the role of direct phosphorylation of GLT-1 protein by PKC, where several independent studies using different models reported that it upregulates [153], has no effect [154], or downregulates GLT-1 protein trafficking to the plasma membrane [155,156,157]. The same controversy was applicable to GLAST, where many research groups had no evidence that PKC phosphorylation influences its plasma membrane trafficking, however, they showed that it reduces its functional activity of glutamate uptake [158,159,160]. A more recent study in 2004 reported that phorbol 12-myristate 13-acetate (PMA, a PKC agonist) increases GLAST surface expression in astroglial cultures, shortly after treatment; however, it has no effect on GLAST with long term treatment [161], while Guillet et al. in 2005 mentioned that PMA decreases GLAST surface expression [157]. The reasons for such variability are still under investigation.

Regarding protein phosphorylation with PKA and P13K, Guillet and colleagues reported that using inhibitors of P13K in neuron-enriched astroglial cultures dramatically reduced GLT-1, but increased GLAST protein trafficking to the cell membrane. In the same study, inhibitors of PKA reduced the cell surface expression of GLAST, while it remarkably increased GLT-1 in the same cultures [157].

Although many research groups identified arachidonic acid (AA) as a general inhibitor of glutamate uptake activity in astrocytes [162,163,164], one study proposed a subtype-specific effect of AA, where it reduces EAAT-1 activity; however, it stimulates glutamate uptake by EAAT-2 [27].

Amyloid β-peptide in patients with Alzheimer’s disease (AD) has been shown to downregulate the functional activity of glutamate transporters [165]; however, in a more recent study, it was reported to increase the cell surface expression of GLAST protein and boost its ability to uptake glutamate [166].

Oxidative stress is one of the major factors that influence the function of glutamate transporters. Two independent research groups demonstrated that H_2_O_2_ significantly reduces glutamate uptake in primary cortical astrocytes and its effect was abolished by treating the cultures with superoxide dismutase and catalase anti-oxidant enzymes [167,168]. This H_2_O_2_-associated suppression of glutamate uptake was due to direct oxidation of the sulfhydryl (SH) group of both transporter proteins [169]. In the same regard, a recent study in 2018 identified ascorbate (antioxidant secreted by astrocytes during glutamate clearance) as an essential antioxidant, protective against neuronal excitotoxicity [170]. In this study, ascorbate-deficient mice experienced behavioral changes and increased susceptibility to seizures compared to wild type (WT) mice. This led the authors to conclude that low levels of antioxidants could explain the development of subclinical seizures associated with the cognitive impairment in patients with AD, who have a remarkably lower level of ascorbate in their CNS [170].

## 3. Glutamate Release by Astrocytes

The interest in astroglial glutamate release developed relatively more recently than glutamate uptake [7]. Cornell-Bell and colleagues reported that glutamate release from excitatory neurons evoked an augmentation of the intracellular Ca^2+^ ([Ca^2+^]_i_) in cultured astrocytes [171]. Later on, two independent research groups showed, in vitro and in vivo, that this [Ca^2+^]_i_ augmentation in astrocytes was followed by a rise in the [Ca^2+^]_i_ of the surrounding neurons [172,173]. Subsequent studies revealed that astrocytic [Ca^2+^]_i_ elevation induced glutamate release from astrocytes [172,174,175]. Interestingly, in addition to glutamate, astrocytes release ATP [176,177], GABA [178,179,180], and d-serine [181] in a process named later as “gliotransmitter release” [182] and these gliotransmitters mediate neuronal excitation or inhibition [183,184,185,186,187,188].

### 3.1. Physiological Role of Astroglial Glutamate Release

Based on recent studies, researchers suggest that glutamate released from astrocytes is involved in the regulation of neuronal activity under physiological conditions (reviewed by Hamilton and Attwell, 2010) [7].

First, astroglial-released glutamate synchronizes excitatory neuronal firing. When glutamate is released from excitatory neurons, it stimulates type I and V metabotropic glutamate receptors (mGluRs) in astrocytes, the activation of which induces elevation of the astrocytic [Ca^2+^]_i_, which in turn triggers glutamate release from astrocytes. The released glutamate activates extra-synaptic *N*-methyl-d-aspartate (NMDA) receptors of the adjacent excitatory neurons, generating slow inward currents inside these neurons that are thought to synchronize their action potential firing [183,184,185,186,189].

Second, glutamate potentiates neuronal excitation. Glutamate released from astrocytes stimulates neuronal presynaptic group I mGluRs or *N*-methyl-d-aspartate (NMDA) receptors, inducing more glutamate release from presynaptic neurons [190,191,192,193].

Third, glutamate potentiates neuronal inhibition. GABA release from interneurons activates GABA_B_ receptors in astrocytes and induces a rise in their [Ca^2+^]_i_ level, which in turn triggers glutamate release. Glutamate acts on neuronal presynaptic ionotropic glutamate receptors (iGluRs) and results in increased GABA release from the surrounding inhibitory neurons [187].

Finally, glutamate potentiates transient hetero-synaptic inhibition in the hippocampus. Glutamate release from the excitatory afferents to CA1 pyramidal cells activates interneurons to release GABA. GABA activates GABA_B_ receptors in astrocytes, elevates [Ca^2+^]_i_ levels, and triggers glutamate release from astrocytes. Glutamate stimulates presynaptic Group I and III mGluRs in the other afferents to inhibit glutamate release [188].

### 3.2. Mechanisms of Glutamate Release by Astrocytes

**Figure 2:**
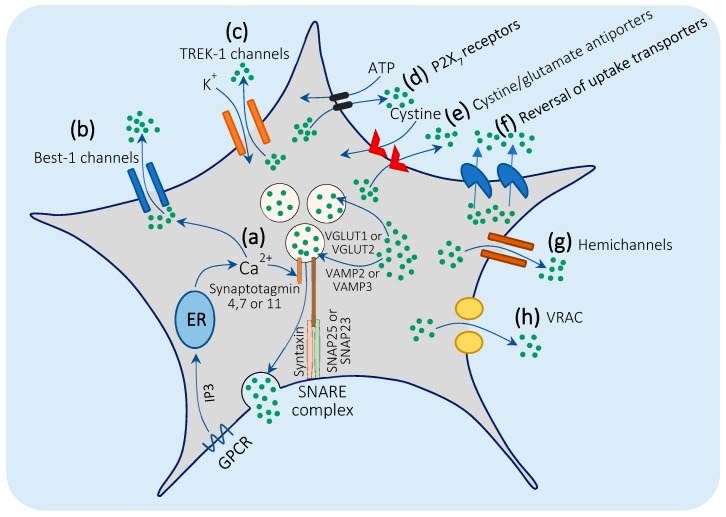
Mechanisms of astrocytic glutamate release: (**a**) Ca^2+^-mediated exocytosis: Astrocytes express VGLUT1 or VGLUT2, which transfer glutamate from the cytosol to the vesicles and vesicular VAMP2 or VAMP3 forms the SNARE complex by binding to syntaxin and SNAP25 or SNAP23 on the cell membrane. Stimulation of GPCRs in astrocytes induces IP3 formation and the release of Ca^2+^ from the ER. This high [Ca^2+^]_i_ concentration is sensed by synaptotagmin 4, 7, or 11, which in turn induces fusion of the vesicles with the cell membrane and triggers glutamate release; (**b**) Best-1-mediated glutamate release: Stimulation of GPCRs in astrocytes, induces glutamate release through Bestrophin-1 channels in a Ca^2+^-dependent mechanism; (**c**) TREK-1 mediated glutamate release: Glutamate release occurs in exchange with K^+^ uptake; (**d**) P2X_7_ ATP receptors: Glutamate is released in exchange with the uptake of ATP; (**e**) cystine/glutamate antiporters: Glutamate is released from astrocytes in exchange with cystine uptake; (**f**) reversal of uptake transporters: Occurs with ATP depletion or reversal of the Na^+^/K^+^ ratio, as in cases of severe ischemia or stroke; (**g**) gap junction hemichannels: Formed by connexin and pannexin proteins that permit the passage of several molecules, including glutamate, from astrocytes to the extracellular space; (**h**) volume-regulated anion channels (VRACs): In case of brain edema, VRACs are activated in swollen astrocytes, in which they open and release glutamate.

Curiously, several mechanisms were identified to be implicated in glutamate release from astrocytes under physiological and pathological conditions (reviewed in detail by Malarkey et al. 2008) [8], including, principally, Ca^2+^-dependent exocytosis, in addition to many other Ca^2+^-dependent or independent mechanisms. Here, we summarize these mechanisms (Figure 2) and the debate that surrounds their potential role in mediating astroglial glutamate release.

#### 3.2.1. Ca^2+^-Mediated Exocytosis

Ca^2+^-mediated exocytosis is thought to be the main proposed mechanism mediating astrocyte glutamate release under physiological conditions (Figure 2a). In fact, evidence suggests that astrocytes express the machinery needed for exocytosis. Hippocampal astrocytes examined by electron microscopy showed small intracellular vesicles (~30 nm), like those found in neurons, from which glutamate could be released [192,194]. Another study, using electrochemical amperometry and frequency-modulated single-vesicle imaging, revealed that cultured astrocytes and freshly cut astrocytes from the rat hippocampus contain large vesicles (~300 nm) that release glutamate in a “Kiss-and run” mode, in which the vesicular membrane rapidly attaches to and detaches from the cell membrane, allowing the vesicles to release only 10% of their glutamate content to the extracellular space [195].

Further studies demonstrated that the small vesicles in astrocytes express vesicular glutamate transporter 1 and 2 (VGLUT1 and VGLUT2) (Figure 2a), and they are situated in astrocytes close to presynaptic neuronal terminals expressing NMDA receptors [194,196]. VGLUT1 and VGLUT2 are believed to transport glutamate from the cytoplasm to the exocytotic vesicles. In neurons, the function of VGLUT1 and VGLUT2 is derived by the voltage and proton gradient generated by the vacuolar (H^+^) ATPase across the vesicular membrane. Similarly, suppressing ATPase in astrocytes blocked Ca^2+^-dependent glutamate release [197,198,199].

In the process of glutamate exocytosis, vesicular soluble *N*-ethylmaleimide-sensitive factor attachment protein receptors (SNAREs) form complexes with the cell membrane SNAREs, releasing glutamate in response to [Ca^2+^]_i_ elevation. SNAREs are proteins on the vesicular membrane and the cell plasma membrane that interact together to induce membrane fusion [7]. In neurons, vesicle-associated membrane protein 2 (VAMP2) attaches to Syntaxin and synaptosomal-associated protein 25 (SNAP25) on the cell membrane to form the SNARE complex. Neurons express the Ca^2+^ sensor, synaptotagmin 1, which senses [Ca^2+^]_i_ elevation caused by Ca^2+^ entry through voltage-gated Ca^2+^ channels, and initiates vesicular fusion to the cell membrane to release glutamate [200]. Interestingly, astrocytes also express VAMP2 [196] or VAMP3 [194], Syntaxin [201], and SNAP25 [202] or its analog, SNAP23 [203], which suggests that they can form the SNARE complex as do neurons. Moreover, further studies demonstrated that astrocytes express synaptotagmin 4, 7, and 11 (analogs of synaptotagmin 1 in neurons) [204,205,206,207], which trigger vesicular glutamate release in response to [Ca^2+^]_i_ elevation in astrocytes (Figure 2a). Noteworthy, [Ca^2+^]_i_ levels must increase to ~250–350 nM to trigger glutamate release from astrocytes [208].

The majority of [Ca^2+^]_i_ elevation necessary for glutamate release by astrocytes comes from the intracellular calcium stores. G protein-coupled receptor (GPCR) stimulation in astrocytes triggers the formation of inositol-1,4,5-trisphosphate (IP3), which, in turn, activates Ca^2+^ release from the endoplasmic reticulum (ER) [7] (Figure 2a). Besides ER, the role of mitochondrial Ca^2+^ cannot be excluded, as Reyes and Parpura revealed that mitochondria play a role in mediating cytoplasmic Ca^2+^ dynamics and, consequently, in Ca^2+^-mediated glutamate release in cultured astrocytes [209]. The entrance of extracellular Ca^2+^ may also play a minor role, as blocking Ca^2+^ entry by Cd^2+^ markedly reduced the mechanically-induced glutamate release from cultured astrocytes [210].

#### 3.2.2. Bestrophin-1 and TREK-1 Channel-Mediated Glutamate Release.

Two new, channel-mediated mechanisms of glutamate release from astrocytes were proposed in 2012 when Woo et al. demonstrated that stimulation of GPCRs in astrocytes triggers glutamate release through two different channels [211]. The first is the Ca^2+^-activated anion channel, Bestrophin 1 (Best 1) (Figure 2b), which releases glutamate that targets synaptic NMDA receptors in neurons [211,212]. However, it is still under investigation whether Best-1 channels can directly sense [Ca^2+^]_i_ elevation [213] or express specific machinery for this purpose. Nevertheless, the neuronal inward currents generated from Best 1-mediated glutamate release were much slower than those generated from glutamate exocytosis [211]. The second glutamate-permeable channel is the Ca^2+^-independent glutamate-permeable, two-pore domain potassium channel, TREK-1 (Figure 2c), which releases glutamate that targets neuronal mGluRs. However, TREK-1-mediated glutamate release is very rapid (within milliseconds) relative to Best-1-mediated release [211].

#### 3.2.3. Glutamate Release through P2X7 Receptors

P2X Purinoceptor 7 (P2X_7_) receptors bind both ATP and glutamate [214]. Activating these receptors in cultured and in situ astrocytes induces ATP uptake and glutamate release simultaneously (Figure 2d) [215]. However, the extent to which these receptors are involved in glutamate release from astrocytes in vivo in healthy conditions remains unknown.

#### 3.2.4. Cystine/Glutamate Antiporters

Cystine uptake transporters are Na^+^-independent, Cl^−^-dependent antiporters that allow glutamate release in exchange of cystine uptake (as explained with glutamate uptake) (Figure 2e). It was reported for the first time in 1999 that the activation of cystine uptake in cerebellar preparations, induced glutamate release from astrocytes and triggered inward currents in the Purkinje cells [216]. Later, similar results were obtained in the rat striatum. When researchers blocked cystine uptake transporters, this resulted in a significant decrease in the extracellular glutamate concentration [217]. Additionally, applying physiological concentrations of cystine, in vivo, to acutely cut brain slices augmented the extracellular glutamate level [218].

#### 3.2.5. Reversal of Glutamate Uptake Transporters

This mechanism occurs, most likely, in pathological conditions only, as blocking glutamate uptake transporters did not influence astrocytic glutamate release in healthy conditions [172,175,219]. In case of ATP depletion or reversal of the Na^+^/K^+^ ratio (reversal of the driving forces of glutamate uptake), as in severe ischemia, glutamate uptake transporters in astrocytes may reverse and start to release glutamate (Figure 2f) [220] in a Ca^2+^-independent mechanism [172,175,219].

#### 3.2.6. Gap Junction Hemichannels

Connexin and Pannexin proteins that form the gap junctions between astrocytes exist in the form of hemichannels that allow the passage of molecules from the cytoplasm to the extracellular space. When these channels were activated in ischemic cultured astrocytes, they were permeable to glutamate (Figure 2g) in a Ca^2+^-independent manner [221,222].

#### 3.2.7. Volume-Regulated Anion Channels (VRACs)

This mechanism of glutamate release was reported, in vitro and in vivo, in swollen astrocytes. As in the case of brain edema and stroke, VRACs open and permit glutamate release to the extracellular space (Figure 2h) [223,224]. It is still uncertain whether the opening of these channels occurs in a Ca^2+^-dependent or independent mechanism [225,226,227].

### 3.3. An Issue of Debate Shrouds Astroglial Glutamate Release

Despite the great efforts that have been done in the last three decades to understand the various mechanisms implicated in astrocytic gliotransmitter release and its significance in the regulation of neuronal activity, it is still an issue of debate whether astrocytes are able to release glutamate, in vivo, under physiological conditions [7,228,229].

Many researchers have several arguments against the ability of astrocytes to release glutamate in physiological conditions. First, the concentration of glutamate in astrocytes is very low compared to neurons, due to the high activity of the enzyme, glutamine synthetase, that converts glutamate to glutamine, and therefore, it is unlikely that astrocytes would be able to preserve glutamate and pack it inside vesicles [230]. Even if they could do so, the concentration of glutamate would be too low to generate these inward currents in the adjacent neurons [231]. Second, all the methods that have been used, till now, to stimulate [Ca^2+^]_i_ elevation (such as drugs, uncaging of Ins(1,4,5)P_3_ or Ca^2+^, and mechanical stimulation of Ca^2+^ release) or to block intracellular Ca^2+^ release (such as Ca^2+^ buffering, TCA cycle inhibition, or tetanus neurotoxin) in astrocytes are non-specific and are not likely to occur in vivo or, at least, one cannot be sure that this released glutamate actually comes from astrocytes in vivo [7].

From our point of view, the recent studies provided satisfactory evidence that astrocytes can release glutamate, in vitro and in vivo. However, many questions remain unanswered, including the principal mechanisms by which astrocytes release glutamate, in vivo, and the contribution of each mechanism of glutamate release to CNS homeostasis under physiological and pathological conditions. How do astrocytes maintain the balance between glutamate uptake and release, and what controls the fate of glutamate in astrocytes? How can neuroinflammation and/or neurodegeneration affect or be affected by glutamate release? All these questions open the door for researchers to generate more hypotheses for in-depth investigations to uncover the mysterious reality of astroglial glutamate release.

## 4. Dysregulation of Astrocytic Glutamate Uptake and/or Release Leads to CNS Disorders

Under normal conditions, glutamate in the extracellular space must be maintained at very low concentrations to prevent overexcitation of glutamate receptors in neurons and protect against neuronal excitotoxicity.

Contrariwise, in CNS disorders, all their pathological mechanisms are accompanied by inflammation. In response to inflammation, astrocytes undergo molecular, morphological, and functional changes in a process known as “reactive astrogliosis,” where reactive astrocytes may lose some of their crucial functions in the CNS or gain many detrimental characteristics that worsen the CNS condition [1]. Therefore, most of the CNS diseases are associated with either loss of astroglial glutamate uptake or excessive gliotransmitter release that predispose to glutamate excitotoxicity [1].

Inhibition of glutamate uptake transporters in astrocytes and, subsequently, impaired glutamate uptake are implicated in the pathogenesis of many CNS pathologies, as in the case of traumatic brain injury, where the expression of both EAAT-1 and EAAT-2 in astrocytes is markedly reduced up to 7 days post-trauma [232], and the same effect on the expression of glutamate transporters occurs in CNS infection with the human immunodeficiency virus (HIV) [233].

Neurodegenerative diseases are also associated with the repression of glutamate uptake [234]. Patients with amyotrophic lateral sclerosis (ALS) suffer from a loss of the motor neuronal functions caused by a lack of EAAT-2 expression in spinal cord astrocytes [235]. Therefore, using β-lactam antibiotics was neuroprotective in the animal models of ALS and brain ischemia, as β-lactam increases the transcription level and functional activity of EAAT-2/GLT-1 in the brain [236]. In addition, impaired glutamate uptake is involved in the pathogenesis of Alzheimer’s disease, Parkinson’s disease, Huntington’s disease, and epilepsy (reviewed in detail elsewhere) [234,237].

Regarding multiple sclerosis (MS), an autoimmune disease affecting the CNS, MOG-activated T cells (myelin-specific T-cells), in vitro, significantly reduced GLAST expression in astrocytic cultures caused by T-cell-mediated release of TNF-α [144]. In another study on the experimental autoimmune encephalomyelitis (EAE, a mouse model of multiple sclerosis), excess extracellular glutamate not only predisposed mice to neuronal death, but also led to degeneration of oligodendrocytes and accelerated demyelination [238]. In human MS as well, many studies provided evidence that glutamate excitotoxicity is implicated in the pathogenesis of the disease [238,239]. Noteworthy, riluzole, an anti-glutamatergic drug, is now in clinical trials for the treatment of recent onset (less than one year) multiple sclerosis [240].

Failure of astrocytes to uptake glutamate is a common feature in CNS disorders associated with depletion or reversal of the driving forces of glutamate uptake. In addition to uptake failure, reversal of uptake transporters may occur in these conditions, leading to excessive release of glutamate, which worsens glutamate excitotoxicity. Brain ischemia or stroke are the best examples of these disorders, as they are associated with intracellular ATP depletion [220,241,242], as well as hepatic encephalopathy, in which intracellular Na^+^ concentration in astrocytes is markedly raised as a result of NH_3_/NH_4_^+^ toxicity [243].

On the other hand, neuronal excitotoxicity caused by excessive Ca^2+^-mediated glutamate release from astrocytes was reported in CNS disorders associated with an excessive release of inflammatory mediators, particularly TNF-α and prostaglandin E [175,219]. These two mediators elevate the [Ca^2+^]_i_ in astrocytes, hence enhancing glutamate exocytosis, as occurs in HIV infection, stroke, AD, and MS [7].

In the same regard, recent studies in the field of neuro-psychiatry proposed that combined dysregulation of both astroglial glutamate uptake and release potentially contributes to the development of mood disorders and depression-like symptoms in animal models, as well as major depressive disorder (MDD) and schizophrenia in humans [237,244,245]. Interestingly, riluzole also mediates an anti-depressant effect in patients with MDD [246,247].

## 5. Mechanism of Glutamate Excitotoxicity

**Figure 3:**
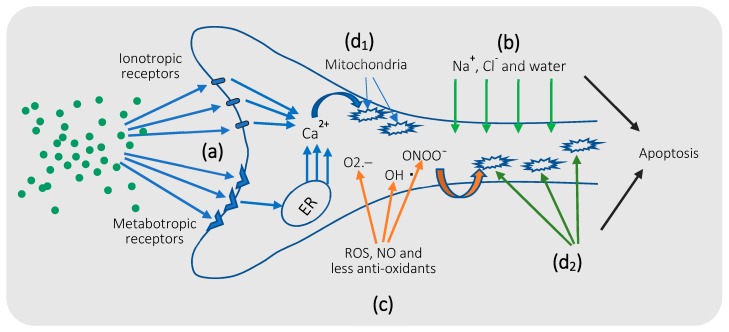
Molecular mechanisms of glutamate excitotoxicity: (**a**) Glutamatergic receptor hyperactivation: Excess extracellular glutamate overstimulates ionotropic (NMDA, AMPA, or KA) and metabotropic (type I and V) receptors in neurons, inducing intracellular Ca^2+^ entry through ionic channels and Ca^2+^ release from the ER, respectively. This raised intracellular Ca^2+^ level represents the first signal for apoptosis; (**b**) excessive ion influx: NMDA receptor hyperstimulation triggers rapid influx of Na^+^, Cl^−^, and water into neurons, resulting in acute rupture of the cell membrane; (**c**) oxidative stress: Excessive production of ROS and NO, and reduced antioxidant mechanisms in neurodegenerative diseases, induce damage to the cellular nucleic acid and intracellular molecules and, eventually, triggers neuronal cell death; (**d**) mitochondrial dysfunction: Overstimulation by elevated [Ca^2+^]_i_ concentration (**d_1_**) and oxidative stress (**d_2_**) trigger the opening of mitochondrial transition pores and release of apoptotic factors to the cytoplasm, which, finally, results in neuronal apoptosis.

Glutamate excitotoxicity is the process by which neuronal death, by apoptosis or necrosis, occurs as a result of excessive or prolonged exposure of neurons to the extracellular glutamate [5]. Different mechanisms interact synergistically and result, eventually, in neuronal death (extensively reviewed by Dong et al. 2009; Wang et al. 2010) [5,248]. Therefore, we summarize the key molecular and cellular mechanisms involved in this process (Figure 3).

First, excess extracellular glutamate, caused by reduced astrocytic glutamate uptake or excessive release, overstimulates three major types of ionotropic glutamate receptors in neurons (Figure 3a), NMDA receptors [249,250,251], α-amino-3-hydroxy-5-methylisoxazole-4-propionate (AMPA) receptors [252], and kainic acid (KA) receptors [253,254]. This ionotropic receptor overstimulation induces excessive intracellular Ca^2+^ entry to neurons through ionic channels. In addition, hyperactivation of mGluRs in neurons, specifically, type I and V mGluRs, induces slower [Ca^2+^]_i_ elevation by coupling to G protein and the generation of IP3, which triggers Ca^2+^ release from the ER [255]. The resulting high intracellular Ca^2+^ levels raise Ca^2+^ concentration in the ER and mitochondria (the Ca^2+^ sensitive organelles), which represents a signal for apoptosis [255]. Moreover, the elevated cytosolic Ca^2+^ level activates calcineurin [256] and/or calpain (apoptotic protease) [257,258] to induce apoptosis.

Second, excessive intracellular Ca^2+^ entry, mediated by NMDA receptor hyperstimulation, triggers a rapid influx of Na^+^, Cl^−^, and water into neurons (Figure 3b), creating an osmotic imbalance that, finally, results in rupture of the cell membrane [259]. Noteworthy, this mechanism commonly induces acute rather than a chronic type of excitotoxic neuronal damage [260].

Third, glutamate excitotoxicity is frequently associated with oxidative stress (Figure 3c), with a high level of intracellular reactive oxygen species (ROS) production [261], commonly, superoxide (O_2_^−^) and hydroxyl (OH.) radicals, that may be associated with downregulation of the anti-oxidant mechanisms in neurodegenerative diseases [262]. Furthermore, Ca^2+^ entry through cation channels after NMDA overstimulation activates nitric oxide synthetase (NOS) enzyme that induces nitric oxide (NO) synthesis. NO may also react with O_2_^−^ to produce peroxynitrite (OONO^−^) [263,264]. Oxidative stress, in turn, causes damage to the intracellular proteins, lipids, and nucleic acid, which activates the intracellular apoptotic pathways [261].

Finally, excessive mitochondrial Ca^2+^ uptake in addition to mitochondrial overstimulation by ROS and NO (Figure 3d) results in the opening of the mitochondrial permeability transition pores and release of pro-apoptotic factors, such as cytochrome C, to the cytosol, which activate mitochondria-mediated apoptotic cascades [265,266].

## 6. Conclusions

Since their discovery, astrocytes were considered as resting cells that fill the space in the CNS, supporting neurons and the BBB. However, recent discoveries on astrocytes motivated researchers to pay more attention to these cells and the vital role they play in modulating neuronal firing, synaptic transmission, and maintaining the homeostasis of the CNS. Instead of looking at astrocytes as passive responders to different CNS pathologies, now, we believe that they are actively implicated in the initiation and progression of many, if not all, CNS diseases.

Knowing the unique role of astrocytes in maintaining glutamate homeostasis and regulating the balance between glutamate uptake and release in the CNS would help us to understand better the mechanisms of the CNS disorders in which glutamate excitotoxicity is involved. It also opens the door for investigators to consider astrocytes as a therapeutic target for these disorders. In this regard, appreciative efforts have already been successful in the field of ALS. A promising astrocyte cell-based therapy, using astrocytes derived from embryonic stem cells, is now in clinical trials following its successful application in the SOD1^G93A^ mouse model of ALS [267,268].
